# Oxygen sensitivity of mitochondrial function in rat arterial chemoreceptor cells

**DOI:** 10.1113/jphysiol.2013.257741

**Published:** 2013-05-13

**Authors:** Keith J Buckler, Philip J Turner

**Affiliations:** Department of Physiology, Anatomy & Genetics Parks Road, Oxford, UK

## Abstract

The mechanism of oxygen sensing in arterial chemoreceptors is unknown but has often been linked to mitochondrial function. A common criticism of this hypothesis is that mitochondrial function is insensitive to physiological levels of hypoxia. Here we investigate the effects of hypoxia (down to 0.5% O_2_) on mitochondrial function in neonatal rat type-1 cells. The oxygen sensitivity of mitochondrial [NADH] was assessed by monitoring autofluorescence and increased in hypoxia with a *P*_50_ of 15 mm Hg (1 mm Hg = 133.3 Pa) in normal Tyrode or 46 mm Hg in Ca^2+^-free Tyrode. Hypoxia also depolarised mitochondrial membrane potential (ψ_m_, measured using rhodamine 123) with a *P*_50_ of 3.1, 3.3 and 2.8 mm Hg in normal Tyrode, Ca^2+^-free Tyrode and Tyrode containing the Ca^2+^ channel antagonist Ni^2+^, respectively. In the presence of oligomycin and low carbonyl cyanide 4-(trifluoromethoxy) phenylhydrazone (FCCP; 75 nm) ψ_m_ is maintained by electron transport working against an artificial proton leak. Under these conditions hypoxia depolarised ψ_m_/inhibited electron transport with a *P*_50_ of 5.4 mm Hg. The effects of hypoxia upon cytochrome oxidase activity were investigated using rotenone, myxothiazol, antimycin A, oligomycin, ascorbate and the electron donor tetramethyl-*p*-phenylenediamine. Under these conditions ψ_m_ is maintained by complex IV activity alone. Hypoxia inhibited cytochrome oxidase activity (depolarised ψ_m_) with a *P*_50_ of 2.6 mm Hg. In contrast hypoxia had little or no effect upon NADH (*P*_50_= 0.3 mm Hg), electron transport or cytochrome oxidase activity in sympathetic neurons. In summary, type-1 cell mitochondria display extraordinary oxygen sensitivity commensurate with a role in oxygen sensing. The reasons for this highly unusual behaviour are as yet unexplained.

**Key points**

- Arterial chemoreceptors measure blood oxygen and are involved in the control of both breathing and the cardiovascular system.
- Oxygen is mostly used by cells in their mitochondria to generate energy.
- In this study we have investigated the effects of oxygen starvation (hypoxia) on the mitochondria of specialised oxygen sensing cells from arterial chemoreceptors.
- Our data confirm that the mitochondria of these oxygen sensing cells are unusually sensitive to modest hypoxia. This effect seems to stem from a reduced affinity of the oxygen utilising enzyme cytochrome oxidase for oxygen.
- These results are consistent with a functional adaptation of the mitochondria of oxygen sensing cells that may enable them to play a direct role in the oxygen sensing process itself.

## Introduction

The ability to sense changes in oxygen plays a vital role in ensuring adequate oxygenation of animal tissues. There are at least two distinct pathways. One involves the control of gene transcription via hypoxia inducible factor, which is regulated by oxygen sensitive prolyl and asparaginyl hydroxylases (Schofield & Ratcliffe, 2004; Kaelin & Ratcliffe, 2008; Semenza, 2012). This pathway responds slowly to sustained hypoxia and is ubiquitous. The other pathway controls cellular activity by regulating ion channels (Weir *et al.* 2005) and is found primarily in a few specialised tissues. This latter pathway can signal acute changes in oxygen within seconds. The archetype of this form of acute oxygen sensing is found in the type-1 cells of peripheral arterial chemoreceptors which drive both ventilatory and cardiovascular reflexes to hypoxaemia. The identity of the sensor involved in acute oxygen sensing is, however, proving to be elusive. One of the earliest, and most controversial, hypotheses is that oxygen sensing in chemoreceptors is linked to oxidative phosphorylation (the metabolic hypothesis). This proposal is supported by a wealth of data demonstrating that arterial chemoreceptors are powerfully excited by all inhibitors of oxidative phosphorylation (Heymans *et al.* 1931; Shen & Hauss, 1939; Anichkov & Belen’kii, 1963; Mulligan *et al.* 1981; Mulligan & Lahiri, 1982; Wilson *et al.* 1994). In the type-1 cell these metabolic poisons elicit a classic pattern of sensory neuronal excitation comprising modulation of ion channels to generate a receptor potential which then stimulates electrical activity, calcium influx and neurosecretion (Obeso *et al.* 1989; Buckler & Vaughan-Jones, 1998; Ortega Saenz *et al.* 2003; Williams & Buckler, 2004; Wyatt & Buckler, 2004; Varas *et al.* 2007). The existence of a metabolic signalling pathway in these cells therefore seems well established; the contentious issue is whether this pathway is the same as that used for acute oxygen sensing.

The standard criticism of the metabolic hypothesis is that cytochrome oxidase has such a high affinity for oxygen that ‘physiological hypoxia’ should have no effect upon mitochondrial energy metabolism. This assertion has rarely been directly tested, but three key studies have reported mitochondrial function in the carotid body to have an extraordinarily high sensitivity to hypoxia (Mills & Jöbsis, 1970, 1972; Nair *et al.* 1986; Duchen & Biscoe, 1992*a*,*b*). Whilst there have been theoretical criticisms of these studies there have been little data obtained from arterial chemoreceptors to countermand them. In this paper we have reinvestigated, and sought an explanation for, the effects of hypoxia upon mitochondrial function in isolated type-1 cells.

Our data fully confirmed previous observations (Duchen & Biscoe, 1992*a*,*b*) that hypoxia increases mitochondrial NADH and depolarises mitochondrial membrane potential (ψ_m_) over a range of 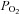 that is both exceptional and commensurate with a role in acute oxygen sensing. We also observed that hypoxia was able to inhibit electron transport and cytochrome oxidase activity at relatively high 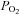 and suggest that this is the most likely cause of changes in NADH and ψ_m_. Possible explanations for the effects of hypoxia on cytochrome oxidase activity and electron transport are considered, but we are as yet unable to give any definitive reason for this unusual behaviour.

## Methods

### Ethical approval

All procedures involving animals were approved by the University of Oxford's Local Ethical Review Process and were conducted in accordance with project and personal licences issued under the UK Animals (Scientific Procedures) Act 1986.

### Cell isolation

Carotid artery bifurcations with attached carotid bodies and superior cervical ganglia were excised from neonatal rats aged 12–15 days under terminal anaesthesia (2–4% halothane in oxygen, at the end of the procedure animals were killed by exsanguination or decapitation). Bifurcations were transferred to ice-cold phosphate-buffered saline and the carotid bodies and/or superior cervical ganglia were dissected free. Type-1 cells were isolated from the carotid body using enzymatic digestion with 0.4 mg ml^−1^ trypsin (Sigma, St Louis, MO, USA) and 0.5 mg ml^−1^ collagenase (Worthington, Lakewood, NJ, USA) in HAMS F-12 for 25–30 min at 35°C followed by transfer to enzyme-free DMEM/F12 culture media and trituration through fire-polished pipettes. The resultant cell suspension was plated on to poly-l-lysine-coated coverslips and maintained in an incubator at 37°C, 5% CO_2_ and 11% O_2_ for between 2 and 8 h before use. Culture media comprised ‘Advanced DMEM/F-12’ (Gibco, Carlsbad, CA, USA) supplemented with 10% heat-inactivated fetal bovine serum, 2 mm l-glutamine and 4 μg ml^−1^ insulin.

Isolation of sympathetic neurons from the superior cervical ganglia used a similar protocol to that described above but with the following modifications: (i) ganglia were cut into 4–5 pieces before incubation in enzyme media and (ii) enzymatic digestion was for 40–50 min.

### Measurement of [Ca^2+^]_i_ and NADH

Fluorescence measurements were performed using a microspectrofluorimeter based on a Nikon Diaphot 200 (Tokyo, Japan) equipped with a xenon lamp to provide an excitation light source and cooled (−20°C) photomultiplier tubes (PMTs; Thorn EMI, London, UK) to detect emitted fluorescence. [Ca^2+^]_i_ was measured using Indo-1 loaded into cells by incubation with 2–5 μm of the acetoxymethyl ester derivative in culture media for 1 h at room temperature. Indo-1 was excited at 340 nm and fluorescence intensity measured at 405 ± 16 nm and 495 ± 10 nm. The fluorescence emission ratio (405/495) for Indo-1 was calibrated as previously described (Buckler & Vaughan Jones, 1993).

**Figure 1:**
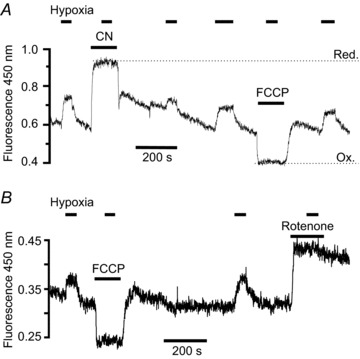
NADH autofluorescence in type-1 cells Recording of autofluorescence at 450 nm with 340 nm excitation from two small clusters of type-1 cells. Note increase in fluorescence in response to electron transport inhibition with 2 mm cyanide (*A*) and 1 μm rotenone (*B*), and decrease in fluorescence with 1 μm FCCP (*A* and *B*). The mitochondrial NAD^+^/NADH couple is assumed to be maximally oxidised in the presence of FCCP, i.e. all in the form of NAD^+^ which is non-fluorescent, and any remaining signal under these conditions is therefore assumed to come from some other source. In the presence of cyanide the mitochondrial NAD^+^/NADH couple is assumed to be maximally reduced, i.e. all in the form of NADH. The difference in fluorescence between the maximally reduced and oxidised states (Red & Ox in *A*) therefore represents total mitochondrial NADH + NAD^+^. Note that hypoxia (2.5% oxygen) increases fluorescence under control conditions but not in the presence of CN, rotenone or FCCP, indicating that hypoxia increases mitochondrial NADH but does not affect other sources of cellular fluorescence.

**Figure 2:**
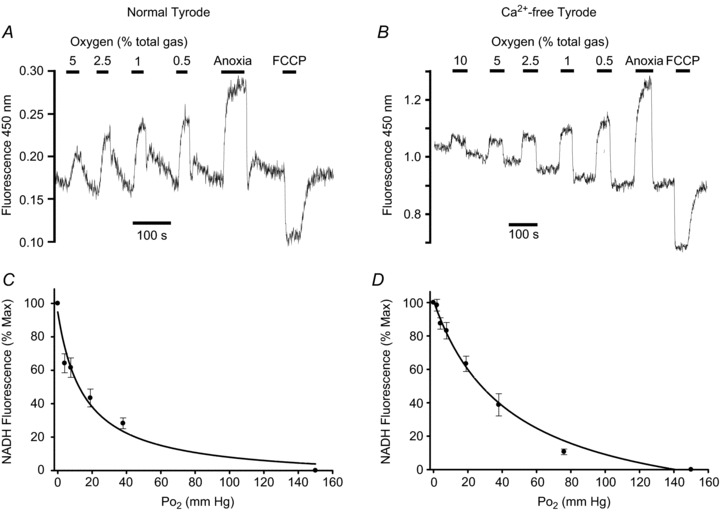
Effects of graded hypoxia on NADH autofluorescence in type-1 cells Recordings of autofluorescence at 450 nm with 340 nm excitation showing effects of graded levels of hypoxia from 5/10% down to anoxia. *A* and *B*, original recordings. *C* and *D*, normalised summary data (mean ± SEM) from 9 (*C*) and 11–17 (*D*) recordings with best fit rectangular hyperbola. The recording and graph on the left (*A* and *C*) were obtained in a normal Tyrode containing 2.5 mm Ca^2+^; the recording and graph on the right (*B* and *D*) were obtained in a Ca^2+^-free Tyrode containing 100 μm EGTA. Note rapid effects of hypoxia at all levels.

**Figure 3:**
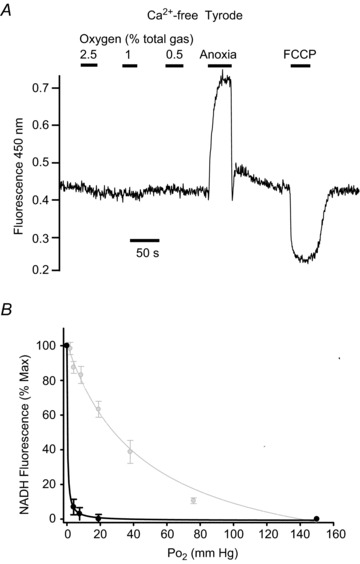
Effects of graded hypoxia on NADH autofluorescence in SCG neuron Recording of autofluorescence at 450 nm with 340 nm excitation showing effects of graded levels of hypoxia from 2.5% down to anoxia. *A*, original recording from a single neuron conducted in Ca^2+^-free Tyrode containing 100 μm EGTA. *B*, normalised summary data (mean ± SEM) from 10 neurons with best fit rectangular hyperbola (black line, see Results for details). Also shown in *B*, in grey, are data and best fit rectangular hyperbola for type-1 cells recorded under identical conditions reproduced from Fig. 2*D*. Note minimal effects of hypoxia even at 0.5%; only anoxia causes a robust increase in NADH autofluorescence in SCG neurons.

NADH was excited at 340 nm and fluorescence measured at 450 ± 15 nm. Raw traces showing fluorescence at 450 nm (NADH) are presented without any correction or calibration. The units shown in the ordinate axis in Figs 1–3 represent the output voltage from an *I–V* converter, with gain 10 nA V^-1^, connected to the PMT. An offset was applied such that 0 (volts) corresponds to the level of background signal in the absence of cells.

### Measurement of mitochondrial membrane potential using rhodamine 123

**Figure 4:**
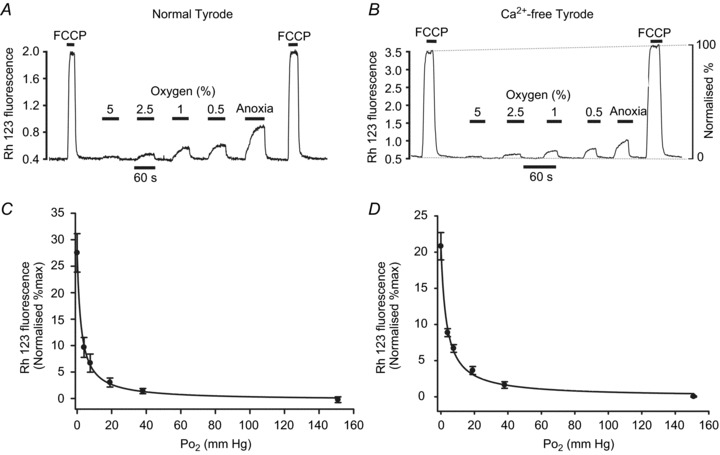
Effects of hypoxia on mitochondrial membrane potential in type-1 cells Measurements of mitochondrial membrane potential were performed using rhodamine 123 (see Methods). *A*, original recording from a small type-1 cell cluster conducted in normal Tyrode. *B*, similar recording obtained in Ca^2+^-free Tyrode containing 100 μm EGTA. Hypoxia ranged from 5% O_2_ down to anoxia. Note application of FCCP (1 μm) causes rapid increase in fluorescence as Rh123 is released from mitochondria due to depolarisation of ψ_m_. Depolarisation is assumed to be complete in 1 μm FCCP. Effects of hypoxia were measured on a 0–100% scale where 0%= interpolated baseline fluorescence in 20% O_2_ and 100%= interpolated maximum fluorescence in 1 μm FCCP. *C* and *D*, normalised summary data (mean ± SEM) from 11 recordings with best fit rectangular hyperbola (see Results for details) for normal Tyrode (*C*) and Ca-free Tyrode (*D*).

**Figure 5:**
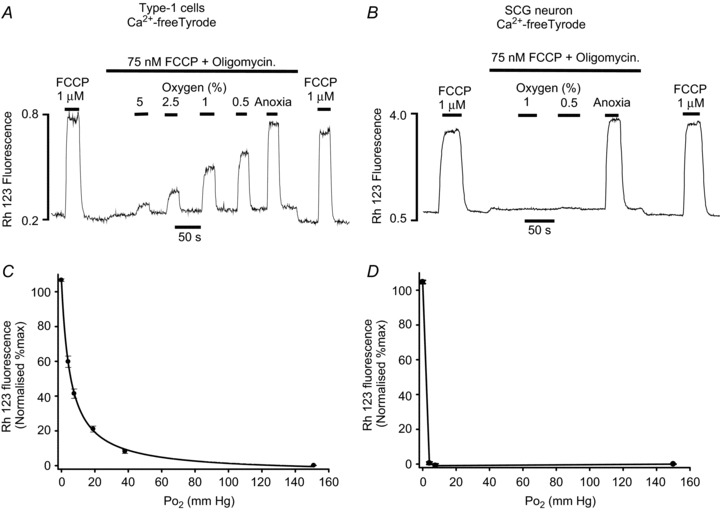
Effects of hypoxia on electron transport Measurements of mitochondrial membrane potential were performed using rhodamine 123. *A* and *B*, original recordings conducted in Ca^2+^-free Tyrode containing 100 μm EGTA. Type-1 cells (*A*) or SCG neurons (*B*) were exposed to a low level of FCCP (75 nm) and oligomycin (2.5 μg ml^−1^) to inhibit ATP synthase and partially uncouple the mitochondria (see text). The effects of hypoxia were then tested in the presence of FCCP and oligomycin. The effect of hypoxia was assessed on a 0–100% scale where 0%= interpolated baseline fluorescence in 20% O_2_, 75 μm FCCP + oligomycin and 100%= interpolated maximum fluorescence in 1 μm FCCP (applied as a brief pulse before and after each experiment). *C*, normalised summary data (mean ± SEM) from 12 recordings obtained in single and small clusters of type-1 cells with best fit rectangular hyperbola (see Results for details). *D*, normalised summary data (mean ± SEM) from 7 recordings obtained in single SCG neurons. Data points in *D* are joined by straight lines only.

**Figure 6:**
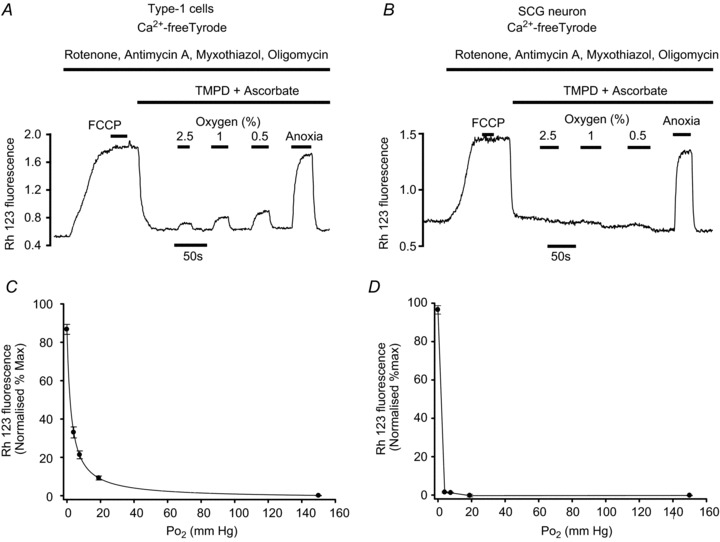
Effects of hypoxia on cytochrome oxidase activity *A* and *B*, original recordings of ψ_m_ performed using rhodamine 123 conducted in Ca^2+^-free Tyrode containing 100 μm EGTA. Type-1 cells (*A*) or SCG neurons (*B*) were exposed first to a cocktail of rotenone, antimycin A, myxothiazol and oligomycin to block complexes I, III and V. This depolarises ψ_m_, which reaches a steady state within about 50 s. Subsequent addition of 1 μm FCCP has no further effect on the Rh123 signal, suggesting that mitochondria are fully depolarised and electron transport is fully inhibited. Addition of TMPD + ascorbate, to provide a source of electrons to cytochrome *c* and thus substrate for cytochrome oxidase, causes a brisk repolarisation of ψ_m_. The effects of hypoxia on ψ_m_, which is assumed to be maintained entirely by cytochrome oxidase activity under these conditions, was then tested. The effects of hypoxia were quantified on a 0–100% scale where 0%= interpolated baseline fluorescence in 20% O_2_+ inhibitors + TMPD/ascorbate and 100%= interpolated maximum fluorescence in 1 μm FCCP (applied as a brief pulse before and after each experiment). *C*, normalised summary data (mean ± SEM) from 14 recordings obtained in single and small clusters of type-1 cells with best fit rectangular hyperbola (see Results for details). *D*, normalised summary data (mean ± SEM) from five recordings obtained in single SCG neurons. Data points in *D* are joined by straight lines only.

Mitochondrial membrane potential (ψ_m_) was monitored using rhodamine 123 (Rh123) in the dequench mode. Rh123 is a fluorescent membrane-permeant cation which passively distributes across membranes according to the membrane potential. When cells are incubated in a solution containing Rh123 it is therefore taken up into the cell and then concentrated within the mitochondria, which have a very negative membrane potential. Under suitable loading conditions Rh123 uptake into mitochondria is associated with partial quenching of its fluorescence (Emaus *et al.* 1986). Once loaded, changes in ψ_m_ cause redistribution of Rh123 between mitochondria and cytosol with consequent changes in the degree of fluorescence quenching; for example, mitochondrial depolarisation causes Rh123 efflux from the mitochondrial matrix into the cytosol resulting in decreased quenching and thus an increase in overall Rh123 fluorescence (Emaus *et al.* 1986; Chen, 1988). Although we cannot directly calibrate Rh123 fluorescence changes in terms of ψ_m_ (see Discussion) we have applied some correction for dye bleaching/leakage. Baseline Rh123 fluorescence (measured under specified conditions) and the maximum fluorescence observed in the presence of 1 μm carbonyl cyanide 4-(trifluoromethoxy) phenylhydrazone (FCCP, a mitochondrial uncoupler) were determined before and after experiments with hypoxia. Linear interpolation was then used to estimate baseline and maximum fluorescence during the intervening recording so that data obtained at any time point could be normalised to a 0% (baseline) to 100% (FCCP) scale. The baseline condition varied depending on the experiment (see Results and figure legends). Raw data traces (Figs 4*A*, *B*, 5*A*, *B* and 6*A*, *B*) are shown without any correction; the units shown in the ordinate axis represent the output voltage from the PMT *I–V* converter (gain 10 nA V^-1^).

### Solutions

Standard bicarbonate-buffered Tyrode solutions contained (in mm): NaCl, 117; KCl, 4.5; CaCl_2_, 2.5; MgCl_2_, 1; NaHCO_3_, 23; glucose, 11. In Ca^2+^-free solutions CaCl_2_ was omitted and 100 μm EGTA added. Normoxic solutions were equilibrated with 5% CO_2_ and 95% air, hypoxic solutions were equilibrated with 5% CO_2_ and 10, 5, 2.5, 1 and 0.5% oxygen (balance nitrogen). Anoxic solutions were produced by equilibration with 5% CO_2_/95% N_2_ followed by the addition of 100–200 μm Na_2_S_2_O_4_ (in experiments involving nickel, Na_2_S_2_O_4_ was added immediately before use). The 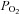 of the control solution was assumed to be 150 mm Hg and that of the anoxic solution to be 0 mm Hg. The 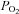 of hypoxic solutions was determined in the recording chamber using a 50 μm fibre optic oxygen sensor (PreSens, Regensburg, Germany) placed close to the bottom of the recording chamber and calibrated using control and anoxic solutions *in situ*. The 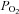 values of the above hypoxic solutions measured 30–60 s after switching from the control perfusate were approximately 76, 38, 19, 8 and 4 mm Hg, respectively. All solutions had a pH of 7.4 at 37°C

### Acquisition and analysis of data

Data acquisition, calibration of the raw data and measurements taken from the raw data were performed using a 1401 interface and Spike 2 software (Cambridge Electronic Design, Cambridge, UK).

Values are expressed as mean ± standard error of the mean (SEM) unless otherwise specified. Statistical analysis was performed using one of the following: (i) a paired two-tailed *t* test for simple comparisons (in Excel, Microsoft), (ii) repeated-measures one-way analysis of variance (RM-ANOVA) with *post hoc* testing against control by the Holm Sidak method (Sigmaplot 12, Sysstat Software Inc, Germany), (iii) RM-ANOVA on ranks with *post hoc* testing against control by the Dunnet method (Sigmaplot 12). Significance was assumed at *P* < 0.05. 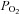 response curves shown in Figs 2–6 are three-parameter hyperbolas of the form *y*=*y*_o_+ (*a*×*b*)/(*b*+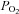) where *b* is the 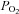 at which a half maximal effect would be observed (*P*_50_). Curve fitting was performed using the non-linear regression dynamic fitting routine in Sigmaplot 12.

### Drugs and chemicals

Antimycin A, myxothiazol, rotenone, tetramethyl- *p*-phenylenediamine (TMPD), FCCP, ascorbate, Rh123 and EGTA were from Sigma; Indo-1-AM was from Invitrogen (Carlsbad, CA, USA); oligomycin was from Calbiochem.

## Results

### NADH autofluorescence

Recordings of cellular autofluorescence were performed upon small clusters of type-1 cells (approx. 2–10 cells) or on single superior cervical ganglion (SCG) neurons. Signal levels were typically low when working with small clusters (2–3) of type-1 cells but could be easily resolved and recordings lasting ≥ 20 min with less than 25% signal loss were readily achievable. A large part of the autofluorescence at these wavelengths is due to mitochondrial NADH; NAD is non-fluorescent and cytosolic NADH levels are normally low. Mitochondrial NADH can be oxidised to NAD by the uncoupling of mitochondrial respiration. Application of 1 μm FCCP caused a rapid reduction in autofluorescence in all type-1 cells/clusters tested to 74% of control (±2.5%, *n*= 5, *P* < 0.005) indicating that approximately 25% of autofluorescence under control conditions was due to mitochondrial NADH (Fig. 1). Just as uncoupling promotes the conversion of mitochondrial NADH to NAD, inhibition of electron transport is expected to promote conversion of mitochondrial NAD into NADH. As expected, application of electron transport inhibitors increased autofluorescence in all type-1 cells tested to 144% (±7.9%, *n*= 6, *P* < 0.05) of control with cyanide (1–2 mm) and 136% (± 1.8%, *n*= 8, *P* < 0.005) of control with rotenone (1 μm). Similar results have also been obtained with other inhibitors of electron transport, including azide (data not shown) and hydrogen sulphide (Buckler, 2012). Uncouplers and electron transport inhibitors can therefore be used to define a range for the mitochondrial NADH signal between conditions of maximal reduction and maximal oxidation (see Fig. 1*A*).

In preliminary experiments we found that superfusing type-1 cells with a moderately hypoxic Tyrode (equilibrated with 2.5% oxygen) also caused a rapid and reversible increase in autofluorescence in all cells/clusters tested (to 123 ± 1.6% of control, *n*= 7, *P* < 0.001). This effect of hypoxia was abolished in the presence of cyanide (Fig. 1*A*, *n*= 4), rotenone (Fig. 1*B*, *n*= 4) and FCCP (Fig. 1, *n*= 4). These data indicate that the increase in autofluorescence during hypoxia is due to an increase in mitochondrial NADH.

We next sought to define the oxygen sensitivity of changes in mitochondrial NADH in type-1 cells. Experiments were conducted by equilibrating normal Tyrode with a range of different gas mixtures containing 0.5, 1, 2.5 and 5% oxygen (all with 5% CO_2_). Measurements of 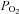 in the recording chamber, made using a 50 μm fibre optic oxygen probe (PreSens), returned values of 3.8, 7.6, 19 and 38 mmHg with each of these gas mixtures, respectively. Anoxic solutions were prepared by equilibrating Tyrode with 5% CO_2_/96% N_2_ for > 10 min followed by addition of 100–200 μm Na_2_S_2_O_4_ before use. Exposure of type-1 cells to hypoxic/anoxic Tyrode resulted in an increase in cellular autofluorescence (*P* < 0.001, *n*= 9, RM-ANOVA) and this was significant at every level of hypoxia tested (*P* < 0.001, *n*= 9, *post hoc* testing by Holm–Sidak method; see Fig. 2*A*). The increase in fluorescence at each level of hypoxia was expressed as a percentage of the increase seen in anoxic solution (relative to control) and is plotted in Fig. 2*C* (data points in anoxia are defined as 100% and those in normal Tyrode (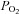
= 150 mm Hg) as 0%). Fitting a rectangular hyperbola (see Methods) to these data returned an *R*^2^ value of 0.84 and a *P*_50_ (the 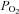 at which a half maximal effect would be observed) of 15 mmHg.

Hypoxia is known to promote Ca^2+^ influx in type-1 cells (Buckler & Vaughan Jones, 1994), elevation of [Ca^2+^]_i_ promotes mitochondrial Ca^2+^ uptake and elevation of mitochondrial [Ca^2+^]_i_ activates NADH producing dehydrogenases (see Discussion). The effects of hypoxia upon NADH could therefore be secondary to these events rather that an inherent property of mitochondrial function. We therefore repeated the above experiments under Ca^2+^-free conditions wherein hypoxia has minimal effect upon cytosolic Ca^2+^ in rat type-1 cells (Buckler & Vaughan Jones, 1994). We performed 17 recordings of NADH autofluorescence in type-1 cells using the above gas mixtures. In 11 recordings we also included a more severe hypoxia (0 oxygen, 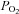
= 2 mm Hg) and in six recordings we included a solution equilibrated with 10% oxygen. Again there was a significant increase in autofluorescence at all levels of hypoxia tested including 10% oxygen (*P* < 0.05, RM-ANOVA with Holm–Sidak *post hoc* testing against control; see Fig. 2*B*). The increase in fluorescence at each level of hypoxia was again expressed as a percentage of the increase seen in anoxic solution (relative to control) and plotted as a function of 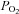 (Fig. 2*D*). Fitting a rectangular hyperbola to these data returned an *R*^2^ value of 0.87 and a *P*_50_ of 46 mm Hg.

As these data suggest an extraordinary degree of oxygen sensitivity in type-1 cells we performed control experiments using another cell type. For this we chose sympathetic neurons from the SCG. These cells were chosen for a number of reasons: (i) the carotid bodies are themselves regarded as part of the sympathetic nervous system (sympathetic paraganglia), (ii) sympathetic neurons are not known to have any oxygen sensing role and (iii) their location in close proximity to the carotid body plus the ability to dissociate neurons from these ganglia using the same enzymatic solutions as those used for dissociating carotid body cells enabled us to ensure that the procedure for preparing isolated SCG neurons was almost identical to that used for type-1 cells (see Methods).

The effects of hypoxia upon autofluorescence in SCG neurons was studied in Ca^2+^-free media as above. Unlike type-1 cells, hypoxia had very little effect upon autofluorescence in SCG neurons whereas anoxia caused a robust increase in autofluorescence, and FCCP caused a robust decrease in fluorescence (Fig. 3*A*). Data obtained in the presence of hypoxia was again converted into a percenatge increase relative to that caused by anoxia. Analysis of these data by RM-ANOVA revealed a significant effect of oxygen overall (*P* < 0.001, *n*= 10), although *post hoc* testing revealed that 2.5% oxygen had no significant effect on fluorescence and that whist there was a statistically significant effect of both 0.5 and 1% oxygen (*P* < 0.05) these effects were very small (7 and 3% of the response to anoxia, respectively). These data are plotted as a function of 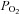 in Fig. 3*B*. A rectangular hyperbola fitted to these data returned an *R*^2^ value of 0.99 and a *P*_50_ of 0.3 mmHg, a value 100-fold less than that obtained in type-1 cells under otherwise identical recording conditions. Data from type-1 cells (from Fig. 2*D*) are reproduced in Fig. 3*B* (in grey) to facilitate comparison with that obtained from SCG neurons.

The significance of these observations is expounded upon further in the Discussion. Suffice to say that our data on type-1 cells are dramatically at variance with conventional wisdom regarding the normal oxygen requirements of mitochondrial respiration but confirm the earlier work of Duchen & Biscoe (1992*a*) on rabbit type-1 cells. We therefore sought to investigate this phenomenon further.

### Mitochondrial membrane potential

Mitochondrial membrane potential was measured using the dye Rh123 in de-quench mode (see Methods). Recordings of ψ_m_ were performed under control conditions and in Ca^2+^-free Tyrode to prevent voltage-gated Ca^2+^ influx during exposure to either hypoxia or FCCP (Buckler & Vaughan Jones, 1998) as Ca^2+^ influx has been shown to depolarise type-1 cell mitochondria (Duchen & Biscoe, 1992*b*). FCCP at 1 μm was applied briefly (for approx. 20 s) both before and after testing the effects of hypoxia upon ψ_m_. Application of FCCP caused a rapid increase in Rh123 fluorescence due to mitochondrial depolarisation and redistribution of Rh123 from mitochondria to cytosol. This response was assumed to represent the maximum (100%) Rh123 fluorescence attainable with complete mitochondrial depolarisation. Measurement of Rh123 fluorescence in the presence of 150 mmHg O_2_ was taken as the minimum level of Rh123 fluorescence (0%). Responses to hypoxia were then quantified on this relative 0–100% scale (see Fig. 4*B*). Hypoxia and anoxia caused a significant increase in Rh123 fluorescence both in the presence (*P* < 0.001, *n*= 11 RM-ANOVA) and in the absence of external calcium (*P* < 0.001, *n*= 11 RM-ANOVA on ranks). This effect was significant (*P* < 0.05 Holm–Sidak *post hoc* test) for all levels of hypoxia in the presence of extracellular calcium and 2.5% oxygen and below in the absence of extracellular calcium (Dunnett's *post hoc* test; note that although data obtained at 5% oxygen failed to reach statistical significance in this test, an increase in Rh123 fluorescence was seen in 10 of the 11 recordings). The increase in fluorescence at each level of hypoxia is plotted as a function of 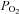 in Fig. 4*C* and *D*. Fitting a rectangular hyperbola to these data returned an *R*^2^ value of 0.96 and a *P*_50_ of 3.1 mmHg in the presence of external calcium and an *R*^2^ value of 0.85 and a *P*_50_ of 3.3 mmHg in the absence of external calcium. Similar results were also obtained in the presence of extracellular calcium and the calcium channel antagonist Ni^2+^, and in a subset of data in normal Ca Tyrode representing recordings from single cells only (see supplemental material).

It is notable that the degree of depolarisation (approx. 22%) in complete anoxia was considerably less than that seen with FCCP despite the fact that anoxia should halt electron transport. In part this may simply be due to the fact that ψ_m_ did not appear to reach a steady state during the brief exposures to anoxia and so our measurement will underestimate the full effect of anoxia. The failure to rapidly and fully depolarise under anoxic conditions is probably due to reversal of the ATP synthase such that ATP is consumed in order to pump protons out of the inner matrix space and help maintain ψ_m_.

### Effects of hypoxia on electron transport

The mitochondrial depolarisation observed in the above experiments could either represent an unusually high degree of oxygen sensitivity in the electron transport chain, or it could be a secondary consequence of increased energy demand leading to increased ATP synthase activity and proton influx into the inner mitochondrial matrix. To evaluate the effects of hypoxia on mitochondrial electron transport alone we sought to isolate ψ_m_ and electron transport from changes in energy demand and the activity of ATP synthase by treating cells with the ATP synthase inhibitor oligomycin. We also increased passive proton leak by applying a low level of uncoupler. Under these conditions ψ_m_ reaches a steady state when the rate of proton translocation by complexes I, II and IV equals passive proton back flux via the uncoupler. Passive proton leak is dependent upon both ψ_m_ and the leak conductance to protons (g_H+_). If we assume that g_H+_ is relatively constant then steady-state ψ_m_ will be linearly related to proton extrusion. Thus, changes in steady-state ψ_m_ will reflect changes in electron transport.

Figure 5*A* shows the protocol employed in these experiments. Cells, bathed in Ca^2+^-free Tyrode throughout, were first exposed to 1 μm FCCP for 20 s to calibrate the Rh123. Cells were then exposed to 75 nm FCCP and 2.5 μg ml^−1^ oligomycin. Oxygen levels in this solution were then reduced, as indicated, in the range 5% to anoxia. Finally, the FCCP/oligomycin solution was removed and a second calibration in 1 μm FCCP performed. Both hypoxia and anoxia caused a brisk and reversible depolarisation in ψ_m_ that reached a steady state within 20 s (*P* < 0.001, *n*= 12, RM-ANOVA). This effect was significant (*P* < 0.001) at all levels of hypoxia tested. These data are plotted as a percentage of maximal depolarisation against 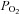 in Fig. 5*C* (taking fluorescence recorded in the presence of 75 nm FCCP and oligomycin in air equilibrated solutions as the baseline, 0%). Fitting a rectangular hyperbola to these data returned an *R*^2^ value of 0.97 and a *P*_50_ of 5.4 mmHg.

The above data indicate that electron transport in type-1 cells would appear to have an unusually high degree of oxygen sensitivity. We therefore repeated the experiment with SCG neurons. The protocol used was identical to that above save for the use of fewer levels of hypoxia (1 and 0.5%). As can be seen from Fig. 5*B* anoxia caused a rapid depolarisation of ψ_m_ to a level comparable to that observed with 1 μm FCCP but little effect was discernible for either of the two hypoxic solutions. The extent of the increase in Rh123 fluorescence as a percentage of that caused by 1 μm FCCP was calculated (as above) and is plotted as a function of 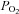 in Fig. 5*D*. RM-ANOVA showed an effect of oxygen on Rh123 fluorescence (*P* < 0.001, *n*= 7) but *post hoc* testing (Holm–Sidak) revealed that this was only significant for anoxia. In view of this we did not attempt to fit a rectangular hyperbola to the data (symbols in Fig. 5*D* are joined by straight lines).

### Oxygen sensitivity of cytochrome oxidase

In their classic paper, Mills & Jöbsis (1972) hypothesised that carotid body cells might contain a cytochrome oxidase with a low oxygen affinity. Such a situation could provide an explanation for the data presented above. We therefore sought to try to assay the oxygen sensitivity of cytochrome oxidase in isolation from the rest of the electron transport chain. To do this we inhibited electron transport complexes I and III and the ATP synthase with a combination of rotenone (1 μm), myxothiazol (0.5 μm), antimycin A (1 μm) and oligomycin (2.5 μg ml^−1^). This cocktail produced a full depolarisation of mitochondria in both Type-1 cells and SCG neurons, i.e. application of 1 μm FCCP had no further effect upon Rh123 fluorescence (see Fig. 6*A* and *C*). Subsequent addition of 5 mm ascorbate plus 40 μg ml^−1^ TMPD, an artificial electron donor capable of reducing cytochrome *c*, resulted in a rapid repolarisation of ψ_m_ (Fig. 6*A* and *C*). Under these conditions ψ_m_ is assumed to be maintained by proton pumping through complex IV alone, with electrons being passed exclusively from TMPD, via cytochrome *c*, through complex IV to oxygen. The effects of reduced oxygen levels on ψ_m_ were then tested under these conditions. As can be seen from Fig. 6*A*, both hypoxia and anoxia caused a depolarisation of ψ_m_ in type-1 cells. These effects were quantified as percentage of maximal depolarisation (in 1 μm FCCP) with the baseline (0%) measured in the presence of all inhibitors plus TMPD and ascorbate in air-equilibrated solutions. These data are plotted as a function of 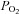 in Fig. 6*B* for type-1 cells and Fig. 6*D* for SCG neurons. Reduction of oxygen level caused a significant depolarisation of ψ_m_ in type-1 cells (*P* < 0.001, *n*= 14, RM-ANOVA). *Post hoc* testing against control (Holm–Sidak method) revealed a significant depolarisation at every level of hypoxia tested (*P* < 0.001). Fitting a rectangular hyperbola to these data returned an *R*^2^ value of 0.95 and a *P*_50_ of 2.6 mmHg. In SCG neurons reducing oxygen also depolarised ψ_m_ (*P* < 0.001, RM-ANOVA, *n*= 5). *Post hoc* testing (Holm–Sidak), however, revealed that only anoxia had a significant effect in SCG neurons. In view of this we did not attempt to fit a rectangular hyperbola to data from SCG neurons; the data points shown in Fig. 6*D* are joined by straight lines only.

### O_2_ sensitivity of mitochondrial function *vs*. chemoreception

**Figure 7:**
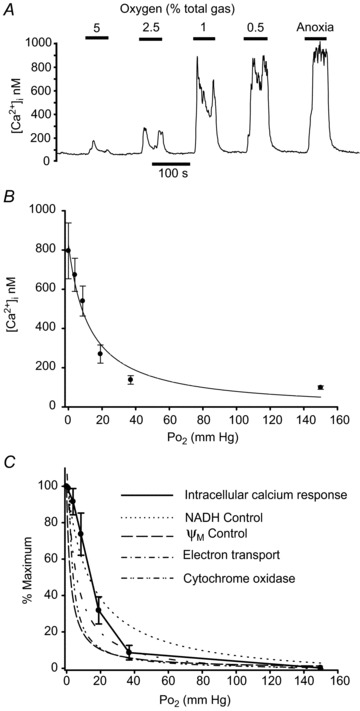
Oxygen sensitivity of calcium signalling and mitochondrial function *A*, original recording of intracellular Ca^2+^ concentration measured using Indo-1 in a small cluster of type-1 cells bathed in normal Tyrode. Cells are exposed to graded hypoxic stimuli from 5% oxygen down to anoxia. *B*, summary data (mean ± SEM) from 11 recordings as in *A*. Curve is a rectangular hyperbola fitted to these data (see text for details). *C*, effects of hypoxia on a relative scale of 0–100% where 0%= baseline [Ca^2+^]_i_ recorded in 20% O_2_ and 100%= maximal [Ca^2+^]_i_ recorded in anoxia. These data (mean ± SEM) are joined by straight lines only. Curves represent oxygen sensitivity of various measures of mitochondrial function in the form of hyperbolic functions fitted to the original data, including: NADH from Fig 2*C*, ψ_m_ from Fig 4*C* (both in normal Ca^2+^ Tyrode), electron transport from Fig. 5*C* and cytochrome oxidase activity from Fig. 6*C*. Hyperbolas have been rescaled, where necessary, to a 0 (baseline in 20% O_2_) to 100% (maximum in anoxia) scale.

One of the objectives of this study was to establish whether mitochondrial respiration in type-1 cells had the requisite oxygen sensitivity to allow it to play a role in acute oxygen sensing. Figure 7 shows measurements of the oxygen sensitivity of calcium signalling in type-1 cells. In this, as in previous studies (Buckler & Vaughan Jones, 1994), hypoxia produced a rapid increase in [Ca^2+^]_i_ in type-1 cells (*P* < 0.001, *n*= 11, RM-ANOVA on ranks). Fitting a rectangular hyperbola to these data returned a value for *R*^2^ of 0.52 and *P*_50_ of 12.5 mm Hg. An equivalent degree of oxygen sensitivity has also been reported for background potassium channel activity in type-1 cells (Buckler, 1997). The [Ca^2+^]_i_ data were normalised, relative to baseline [Ca^2+^]_i_ in air/CO_2_-equilibrated saline (0%) and maximum [Ca^2+^]_i_ in anoxia (100%), and are re-plotted in Fig 7*C*. The oxygen sensitivity of type-1 cell NADH, ψ_m_, electron transport and cytochrome oxidase activity are also plotted in Fig. 7*C*, for comparison.

## Discussion

Our data suggest that modest hypoxia inhibits electron transport in type-1 cells and confirm earlier observations regarding the unusual oxygen sensitivity of mitochondrial function in these cells (Duchen & Biscoe, 1992*a*,*b*). Before discussing the significance of these findings we first consider whether changes in ψ_m_ or NADH are open to alternative interpretation.

Under normal conditions hypoxia and metabolic inhibitors cause voltage-gated Ca^2+^ influx in type-1 cells (Buckler & Vaughan Jones, 1994; Wyatt & Buckler, 2004), which could result in Ca^2+^ uptake by mitochondria (Friel & Tsien, 1994; Werth & Thayer, 1994; Herrington *et al.* 1996) depolarising ψ_m_ and activating Krebs cycle dehydrogenases generating NADH (McCormack & Denton, 1989). Measurements of NADH in Ca-free media and ψ_m_ in both Ca-free media and in the presence of the Ca-channel antagonist Ni^2+^, demonstrate that the effects of hypoxia on NADH and ψ_m_ are not a consequence of cytosolic Ca^2+^ signalling.

Changes in ψ_m_ could occur if mitochondria were tightly coupled with low energy demand (state 4) such that ψ_m_ reached the limit imposed by the free energy available from NADH oxidation (which is dependent upon oxygen concentration). Alternatively, an increase in energy demand could increase ATP-synthase activity and proton influx (i.e. a switch to state 3 respiration). To address these issues we performed experiments with ATP synthase blocked and mitochondria partially depolarised by a low concentration of uncoupler. Again, however, relatively mild hypoxia decreased ψ_m_ in type-1 cells. We assume that with ATP synthase blocked ψ_m_ reaches a steady state when proton efflux via complexes I, III and IV matches proton influx via passive leak (presumed to be predominantly via FCCP). Since passive proton influx must decrease as ψ_m_ depolarises, a decline in steady-state ψ_m_ in hypoxia indicates a corresponding decrease in proton efflux by the electron transport chain. These data therefore confirm that hypoxia can limit electron transport even in partially depolarised mitochondria and independently of ATP synthesis.

### Oxygen sensitivity of mitochondrial function in type-1 cells *vs*. other tissues

Studies of mitochondrial function in other tissues using respirometry indicate a very high affinity for oxygen typically in the range 0.08–0.75 mm Hg (Gnaiger *et al.* 1998; Scandurra & Gnaiger, 2010). Measurements of cytochrome *c* redox state, however, show a much greater sensitivity to oxygen with cytochrome *c* becoming reduced at higher levels of 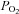 than those required to inhibit oxygen consumption (Wilson *et al.* 1979*a*). Indeed this rise in electron transport chain (ETC) intermediates/cytochrome *c* is thought to partially offset the effects of a fall in oxygen so as to help maintain electron transport rate under conditions of moderate hypoxia (Wilson *et al.* 1979*b*). Defining what constitutes the oxygen sensitivity of mitochondrial function is therefore not entirely straightforward as changes in the levels of intermediates may be more sensitive to 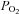 than electron flux. This effect can be seen in our data when comparing the oxygen sensitivity of [NADH] with measurements that reflect electron transport rate (ψ_m_), the former appearing to be more oxygen sensitive than the latter. The most striking feature of our data, however, is that the *P*_50_ values for these effects are at least an order of magnitude greater than those reported in other tissues. This leads us to consider whether there could be a major systematic error in our measurements.

One potential source of error is the accuracy with which tissue oxygen can be controlled and measured. We have used single cells and small clusters of cells (2–8 cells) dispersed at very low density (<100 cells mm^−2^). Rat type-1 cells are small (diameter approx. 10–11 μm) so we assume there is no significant diffusion gradient within individual cells or small clusters. Our perfusion system has rapid solution exchange (half time 1–2 s) such that oxygen levels stabilise within 30 s at 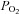 values very close to those expected (see Methods). Significant overestimation of the oxygen levels to which cells were exposed is therefore highly unlikely and measurements of mitochondrial function are made under steady-state conditions with respect to 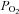.

Another potential source of error is in the calibration of fluorescent signals. NADH autofluorescence bleaches slightly during experiments; as a consequence of this, and applying hypoxic solutions in a decrementing sequence (from high 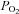 to low 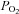), there is likely to be systematic overestimation of the effects of higher *vs*. lower 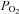 and consequently *P*_50_ values will probably also be overestimated. We have calibrated Rh123 fluorescence using a 0–100% scale. A linear relationship between Rh123 fluorescence and ψ_m_ has been reported for mitochondria studied in a cuvette (Emaus *et al.* 1986) but it is by no means certain that the same will apply in a small cell. Indeed if Rh123 distributes between mitochondria and cytoplasm according to the Nernst equation one might expect the relationship between fluorescence and ψ_m_ to be non-linear particularly at very negative potentials such that changes in Rh123 fluorescence could underestimate the true change in ψ_m_. It should also be noted that we have implicitly assumed a uniform affinity for O_2_ (*P*_50_) by fitting data to a simple hyperbolic function. This does not allow for the possibility of low- and high-affinity forms of cytochrome oxidase as suggested by Mills & Jöbsis (1972). Our estimation of the oxygen sensitivity (*P*_50_) of NADH, ψ_m_, electron transport and cytochrome oxidase can therefore only be considered approximate. These errors are, however, unlikely to account for differences in *P*_50_ of 1–2 orders of magnitude. Moreover, none of these errors could account for the robust changes in mitochondrial function we have observed at modest levels of hypoxia. The best test for methodological errors, however, lies in the use of another cell type. Measurements of the effects of hypoxia on NADH in SCG neurons returned a value for *P*_50_ that was two orders of magnitude lower than that seen in the carotid body. Similar measurements of the effects of hypoxia on electron transport and cytochrome oxidase activity in SCG neurons were unable to resolve a value for *P*_50_ as we were unable to generate 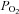 levels low enough to inhibit either electron transport or cytochrome oxidase activity (save for complete anoxia, which is generated with the aid of an oxygen scavenging agent). Despite this technical failure it is evident that whatever the true oxygen sensitivity of electron transport and cytochrome oxidase activity in the SCG neuron, *P*_50_ values must be well below those seen in type-1 cells. In conclusion, we cannot account for our data in any way other than to propose that the apparent oxygen affinity for mitochondrial respiration in type-1 cells is lower than that seen in other cell types by up to two orders of magnitude.

### Mechanisms for reduced oxygen affinity in cytochrome oxidase

To evaluate the oxygen sensitivity of cytochrome oxidase we employed artificial electron donors (TMPD and ascorbate) to maintain a supply of reduced cytochrome *c* and measured the effects of oxidase-mediated proton efflux upon ψ_m_ under conditions in which complexes I, III and IV are blocked. Under these conditions we again saw that hypoxia depolarised ψ_m_, indicating inhibition of cytochrome oxidase activity (Fig. 6*A* and *C*).

Why cytochrome oxidase in type-1 cells should appear much more sensitive to hypoxia than in other cells is unknown. Since there is no precedent for this we can only offer conjecture. The suggestion that there is an alternative low-affinity form of cytochrome *a*_3_ (Mills & Jöbsis, 1972) seems unlikely as there is only one, mitochondrial, gene for subunit I of cytochrome oxidase (although there must remain the possibility of some form of post-translational modification). It is, however, the *apparent K*_m_ that defines oxygen sensitivity of electron transport and this can be influenced by other factors. The high apparent affinity of cytochrome oxidase for oxygen is dependent upon kinetic trapping by electron transfer from the low spin haem to the iron–copper binuclear centre where oxygen is bound (the initial binding of oxygen being weak). As a consequence, the operational *K*_m_ is dependent upon the rate of electron transfer and kinetic trapping (Verkhovsky *et al.* 1996). The oxygen sensitivity of cytochrome oxidase could therefore be influenced by other components of complex IV.

The apparent affinity of cytochrome oxidase could also be lowered through the presence of an endogenous competitive inhibitor. Nitric oxide can inhibit mitochondrial respiration with high affinity, i.e. in the nanomolar range (Brown & Cooper, 1994; Cooper & Giulivi, 2007). Knockout of neuronal nitric oxide synthase (nNOS), however, augments chemoreceptor responses to hypoxia (Kline *et al.* 2002). Knockout of enothelial NOS (eNOS) on the other hand blunts chemoreceptor oxygen sensitivity (Kline *et al.* 2000), yet oxygen sensitivity remains in isolated type-1 cells from control animals (so oxygen sensitivity is largely endothelium independent). CO can also inhibit cytochrome oxidase but its affinity is low (Cooper & Brown, 2008). Indeed, very high levels (>300 mmHg, approx. 280 μm) of exogenous CO are required to excite the carotid body via inhibition of cytochrome oxidase, an effect that is reversed by photo-dissociation (Lahiri *et al.* 1993*a*; Wilson *et al.* 1994). It seems unlikely that endogenous CO could be maintained at such levels moreover the response to hypoxia is not light sensitive (Lahiri *et al.* 1993*a*; Wilson *et al.* 1994).

Finally, we consider the possibility that hypoxia inhibits cytochrome oxidase via another oxygen sensing pathway either as an epiphenomenon or as an integral part of the O_2_ signal transduction pathway. It has recently been suggested that endogenous H_2_S production mediates acute oxygen sensing in blood vessels (Olson *et al.* 2006; Olson & Whitfield, 2010), fish gill chemoreceptors (Olson *et al.* 2008) and the mammalian carotid body (Peng *et al.* 2010; Olson, 2011) with H_2_S levels increasing during hypoxia (Olson *et al.* 2006). Knockout of one of the enzymes that can generate H_2_S appears to blunt chemoreceptor responses to hypoxia (Peng *et al.* 2010; Olson, 2011). H_2_S is a non-competitive inhibitor of cytochrome oxidase (Cooper & Brown, 2008). Micromolar levels of exogenous H_2_S inhibit mitochondrial metabolism and excite type-1 cells (Buckler, 2012). There is, however, uncertainty about whether endogenous H_2_S could reach such levels in isolated cells *in vitro* (Buckler, 2012).

There are therefore potential explanations for the unusual oxygen sensitivity of mitochondrial respiration in this tissue although there are uncertainties about the feasibility of some of them. Clearly more research is needed to identify the causes of this phenomenon.

### Role of mitochondria in oxygen sensing

It is clear from the above discussion that mitochondrial function in type-1 cells is extraordinarily oxygen sensitive, particularly with respect to changes in NADH levels. This degree of oxygen sensitivity is not dissimilar to that of calcium signalling in type-1 cells (Fig. 7*C*). Indeed under some conditions the oxygen sensing range may even extend well into hyperoxia given that we were able to demonstrate changes in NADH between 150 and 75 mm Hg whereas normal carotid body tissue 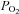 is unlikely to be greater than 60 mmHg (Lahiri *et al.* 1993*b*). While we cannot completely exclude the possibility that this is an epiphenomenon driven by some other unknown O_2_ sensor, it is difficult to reconcile our data with any single effect other than an inhibition of electron transport. Since all other interventions which inhibit electron transport also cause excitation in the carotid body, the most parsimonious explanation of all these data is that even if complex IV is not the primary site of oxygen sensing, modulation of electron transport is probably an integral part of the hypoxia transduction cascade. We have not examined the link between hypoxic inhibition of electron transport and chemoreceptor excitation in this study, but previous studies using a variety of chemical inhibitors of mitochondrial function indicate that inhibition of ATP synthesis appears to be the key event (Wyatt & Buckler, 2004). Changes in cytosolic nucleotide levels may then be detected by TWIK-related acid-sensitive potassium channels (TASK) directly and/or via an AMP kinase (Varas *et al.* 2007; Wyatt *et al.* 2007).

Whilst mitochondrial/metabolic signalling pathways could not account for all forms of acute oxygen sensing, e.g. that reported for ion channels in excised patches (Ganfornina & Lopez Barneo, 1991; Riesco Fagundo *et al.* 2001), it is of interest to note that mitochondria have been implicated in acute oxygen sensing in several other tissues, including adrenal chromaffin cells and pulmonary arterioles (Mojet *et al.* 1997; Buttigieg *et al.* 2008; Ward, 2008; Sylvester *et al.* 2012). The pathways involved in these tissues are various and do not necessarily depend upon metabolic signalling per se; changes in mitochondrial superoxide production have also been suggested (Archer *et al.* 1993; Waypa *et al.* 2001). It will nonetheless be interesting to see if the oxygen sensitivity of mitochondrial function in these other tissues is similarly enhanced.

## Abbreviations

ψ_m_: mitochondrial membrane potential
ETC: electron transport chain
FCCP: carbonyl cyanide 4-(trifluoromethoxy) phenylhydrazone
PMT: photomultiplier tube
Rh123: rhodamine 123
SCG: superior cervical ganglion
TASK: TWIK-related acid-sensitive potassium channel
TMPD: tetramethyl-*p*-phenylenediamine
