# Supplementary material for: Oxygen sensitivity of mitochondrial function in rat arterial chemoreceptor cells
Source: J Physiol. 2013 May 13;591(Pt 14):3549–63. doi: 10.1113/jphysiol.2013.257741 (PMC3731613; doi:10.1113/jphysiol.2013.257741)
Supplement: Supplementary file 1 [file tjp0591-3549-SD1.pdf]

Supplementary Figure 1.

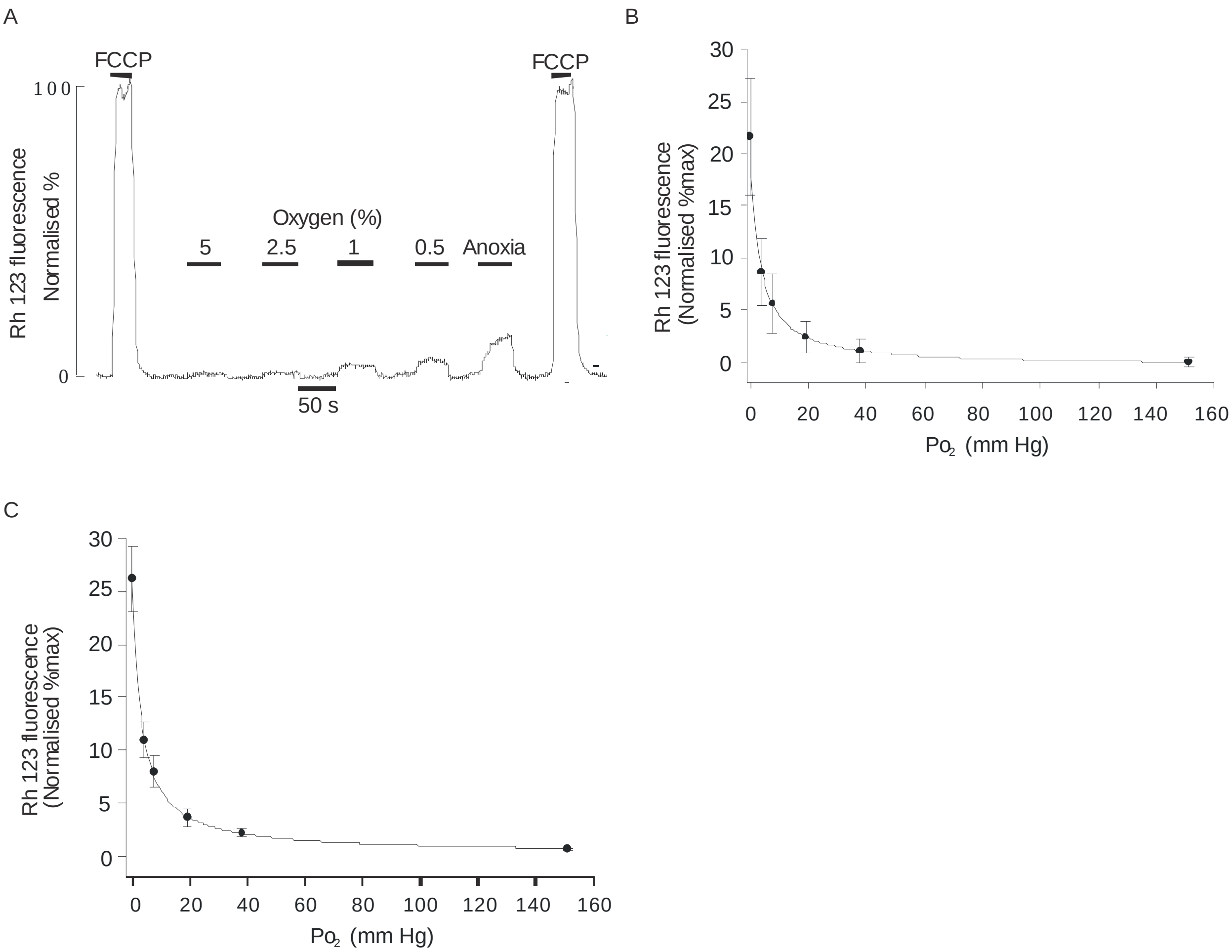

Figure 1. Effects of hypoxia on mitochondrial membrane potential.

Measurements of mitochondrial membrane potential were performed using rhodamine 123. (**A**) Shows a recording of normalised rhodamine 123 fluorescence from a pair of type-1 cells conducted in Ca^{2+}-containing Tyrode plus 1 mM $NiCl_2$. (**B**) Shows normalised summary data for experiments conducted as in A with 1 mM $NiCl_2$. Effects of hypoxia were measured on a 0-100% scale where 0% = interpolated baseline fluorescence in 20% O_2 and 100% = interpolated maximum fluorescence in 1 µM FCCP. Data are mean (± SEM) from 11 recordings. Hypoxia and anoxia caused a significant increase in Rh123 fluorescence ($P < 0.001$, n=11 RM-ANOVA on ranks). Fitting a rectangular hyperbola to this data returned an R^2 value of 0.86 and a P_{50} of 2.8 mmHg. (**C**) Shows normalised summary data for experiments conducted on single type-1 cells in the presence of normal extracellular calcium. Data are mean (± SEM) from 6 recordings. Fitting a rectangular hyperbola to this data returned an R^2 value of 0.97 and a P_{50} of 2.9 mmHg.
